# Retraction: Modulation by miR-29b of intestinal epithelium homoeostasis through the repression of menin translation

**DOI:** 10.1042/BCJ20141028_RET

**Published:** 2026-05-21

**Authors:** Jian-Ying Wang, Miao Ouyang, Weijie Su, Lan Xiao, Jaladanki N. Rao, Liping Jiang, Yanwu Li, Douglas J. Turner, Myriam Gorospe

**Affiliations:** Cell Biology Group, Department of Surgery, University of Maryland School of Medicine, Baltimore, MD, U.S.A.

**Keywords:** intestinal epithelial cell, intestinal epithelium homoeostasis, menin, microRNA, post-transcriptional regulation

This article is being retracted from the *Biochemical Journal* at the request of the Editor-in-Chief and the Editorial Board. This follows the receipt of a notification from a reader, alerting the Editorial Board to multiple similarities between this article and another published by the same author group: Cui et al. 2012 (DOI: 10.1091/mbc.E11-05-0456). Specifically, these include similarities between:

- The Figure 7A Scramble/No-TNFa/CHX panel of this paper and the Figure 7A Control/No-TNFa/CHX panel from Cui et al. 2012.
- The Figure 7A Pre-miR-29b/No-TNFa/CHX panel of this paper and the Figure 7A Anti-miR-503/No-TNFa/CHX panel from Cui et al. 2012.
- The Figure 7A Pre-miR-29b/TNFa-CHX panel of this paper and the Figure 7A Anti-miR-503/TNFa-CHX panel from Cui et al. 2012.
- The Figure 7A Pre-miR-29b + Vector/No-TNFa-CHX panel of this paper and the Figure 7A Anti-miR-503 + C-siRNA/No-TNFa-CHX panel from Cui et al. 2012.
- The Figure 7A Pre-miR-29b + Vector/TNFa-CHX panel of this paper and the Figure 7A Anti-miR-503 + C-siRNA/TNFa-CHX panel from Cui et al. 2012.
- The Figure 7A Pre-miR-29b + MEN1OE/No-TNFa-CHX panel of this paper and the Figure 7A Anti-miR-503 + siCUGBP1/No-TNFa-CHX panel from Cui et al. 2012.

The Editorial Office also notes that the two Figure 3C AHA-GAPDH blots of this article are highly similar to each other.

The authors were contacted regarding the concerns raised and provided a replacement for Figure 7A, along with raw data. No raw data were able to be provided for the Figure 3C western blots. The Editorial Board also has concerns over the new raw data provided for Figure 7A, prompting the decision to retract. As such, the Editorial Board stands by the decision to retract. The authors disagree to the retraction.

